# Is IORT ready for roll-out?

**DOI:** 10.3332/ecancer.2015.516

**Published:** 2015-03-12

**Authors:** Emanuela Esposito, Bauke Anninga, Ian Honey, Gillian Ross, Dick Rainsbury, Siobhan Laws, Sygriet Rinsma, Michael Douek

**Affiliations:** 1 Research Oncology, Division of Cancer Studies, King’s College London, Guy’s Hospital, Great Maze Pond, London SE1 9RT, UK; 2 Department of Breast Surgery, Istituto Nazionale per lo Studio e la Cura dei Tumori ‘Fondazione Giovanni Pascale’ – IRCCS, Naples 80131, Italy; 3 Department of Clinical Medicine and Surgery, Breast Unit, University of Naples Federico II, Naples 80131, Italy; 4 Department of Medical Physics Floor 3, Henriette Raphael House, Guy’s and St Thomas Hospital, London SE1 9RT, UK; 5 Clinical Oncology, Royal Marsden Hospital and Institute of Cancer Research, London SW3 6JJ, UK; 6 Oncoplastic Breast Unit, Royal Hampshire County Hospital, Winchester SO22 5DG, UK

**Keywords:** breast cancer, intraoperative radiotherapy, IORT

## Abstract

Two large randomised controlled trials of intraoperative radiotherapy (IORT) in breast-conserving surgery (TARGIT-A and ELIOT) have been published 14 years after their launch. Neither the TARGIT-A trial nor the ELIOT trial results have changed the current clinical practice for the use of IORT. The in-breast local recurrence rate (LRR) after IORT met the pre-specified non-inferiority margins in both trials and was 3.3% in TARGIT-A and 4.4% in the ELIOT trial. In both trials, the pre-specified estimates for local recurrence (LR) with external beam radiation therapy (EBRT) significantly overestimated actual LRR. In the TARGIT-A trial, LR with EBRT was estimated at the outset to be 6%, and in the ELIOT trial, it was estimated to be 3%. Surprisingly, LRR in the EBRT groups has been found to be significantly lower, 1.3% in the EBRT arm of the TARGIT-A and 0.4% in the EBRT arm of the ELIOT trial, respectively. Median follow-up was 2.4 years for the TARGIT-A trial and 5.8 years for the ELIOT trial. However, the initial cohort of patients in the TARGIT-A trial (reported in 2010) now have a median follow-up of 3.8 years and data on LR were available at 5 years follow-up on 35% of patients (18% who received IORT). Although further follow-up will increase confidence with the data, it will also further delay clinical implementation. By carefully weighing the risks and benefits of a single-fraction radiation treatment with patients, IORT should be offered within agreed and strict protocols. Patients deemed at low risk of LR or those deemed suitable for partial breast irradiation, according to the GEC-ESTRO and ASTRO recommendations, could be considered as candidates for IORT. These guidelines apply to all partial breast irradiation techniques, and more specific guidelines for IORT would assist clinicians.

## Background

External beam radiation therapy (EBRT) following breast-conserving surgery (BCS) is the standard of care for the treatment of early-stage breast tumours. It has been shown to reduce local recurrence (LR) and improve overall survival [1–4]. Women undergoing post-operative EBRT are required to attend the radiation treatment 5 days a week for 3–5 weeks consecutively. Almost one-third of patients who undergo BCS for early breast cancer in North America do not receive post-operative breast irradiation because they live far from a treatment centre and cannot attend daily RT [5, 6]. This issue affects patients suffering from breast cancer all over the world and is particularly challenging for the elderly people. In some countries, patients decline RT or even choose mastectomy in order to avoid EBRT [7–9]. Consistent with this, IORT and some other shortened and localised radiotherapy treatments were evaluated.

Moreover, recently, the expansion of oncoplastic breast surgery allows BCS to be offered to patients in whom a wide local excision (WLE) would have a poor cosmetic result but provides a challenge for the subsequent radiation planning, particularly for the delivery of the tumour bed boost. The target volume may be partially missed by externally delivered boost, especially with tissue displacement techniques [10]. IORT can be delivered prior to tissue displacement and a direct visualisation of the tumour bed can guarantee an accurate dose delivery. The growing body of scientific evidence, in which IORT was given as a boost, has provided outstandingly low local recurrence rates [11, 12].

## The rationale for IORT

Several clinical observations led to an interest in local radiation treatment following BCS. Firstly, it is observed that over 90% of LR after BCS occur at or near the original operation site [13–15]. Secondly, the risk of ipsilateral new breast primaries is equal to the risk of contralateral breast cancer [16–18]. Lastly, if whole EBRT is used to reduce the occurrence of new breast primaries, then this same principle should apply to the contralateral breast. In an effort to offer both shortened and more localised RT regimens, partial breast irradiation (PBI) that directly targets the index quadrant has been under clinical investigation. PBI treatments include multicatheter brachytherapy, intracavitary brachytherapy, three-dimensional conformal radiotherapy (3D-CRT), stereotactic body radiation therapy (SBRT) [19, 20], and intraoperative radiotherapy (IORT) [21–25]. Among the various PBI techniques, the largest randomised controlled trials were conducted on IORT.

Several single institution cohort studies have been published on IORT, but only two phase-III trials have been launched and completed recruitment, the targeted intraoperative radiotherapy-alone (TARGIT-A) trial [26] and the electron intraoperative treatment (ELIOT) trial [27]. One of the most important advantages of IORT is the delivery of a single fraction of RT at the time of surgery, directly into either the tumour cavity or the index quadrant. By employing IORT, waiting times for EBRT are reduced, and departments can focus on more complex radiotherapy treatments, since over 30% of the workload of RT departments constitutes EBRT for breast cancer. On the other hand, a disadvantage of IORT is the longer operating time required, the equipment cost, the service cost, and the cost of the additional staffing.

## Available devices and techniques for IORT

The available methods of delivering IORT are low-energy X-ray systems [26], electron beam radiation therapy [27], high dose rate after loaders [28] or specific balloon devices [29]. Low-energy X-ray IORT is delivered by the Intrabeam^®^ device (Carl Zeiss, Oberkochen, Germany). It is a miniature low-energy X-ray source employed for IORT during surgery after removal of the tumour. With the Intrabeam^®^ device treatment time ranges from 20 to 40 minutes, delivered dose is 20 Gy at the surface of the applicator and 5–6 Gy at 1 cm depth. Tungsten-impregnated sheets are used to shield the wound prior to treatment. These block 95% of radiation, but radiation doses within the operating room remain potentially significant and necessitate control of access to the room during treatment and further shielding for the anaesthetist and medical physicists. Existing walls will often provide sufficient shielding for the low-energy X-rays, and thus, it is often possible to use existing operating rooms. Before introducing the Intrabeam^®^ system, it is thus important to consider control of access to the operating room, to undertake a shielding assessment and to measure environmental radiation dose rates around the theatre.

Electron beam radiation therapy (EBRT) or intraoperative electron radiation therapy (IOERT) is the application of electron radiation directly to the tumour bed at the time of surgery. Electron beams can deliver the required dose much more rapidly than other devices. Mostly, to treat breast tumours, it has been estimated that 12 MeV energy is sufficient. These systems are designed with the concept of being utilised to deliver radiation in non-shielded operating rooms and are provided with a beam stopper. The beam stopper for certain mobile IORT units is designed to track the movement of gantry in all directions so that it will always intercept the primary beam, whereas other beam stoppers must be manually positioned. Three mobile linear accelerators can deliver IOERT, the Novac7^®^ (Hitesys S.p.A., Aprilia, Latina, Italy), the Liac^®^ (Sordina, Padova- Italy), and the Mobetron^®^ (IntraOP Medical Inc, Sunnyvale, CA). Both Novac7^®^ and Liac^®^ have been employed in a phase-III trial, the ELIOT trial. The entire irradiation procedure is completed in 2 minutes, and the delivered dose is 21 Gy with the depth of 90% isodose ranging from 13 (3 MeV) to 24 mm (9 MeV).

High-dose-rate (HDR) afterloader (Mick Radio-Nuclear Instruments, Inc., Mount Vernon, NY) within a dedicated shielded operating facility (Brachytherapy Unit) was evaluated by Memorial Sloan–Kettering Cancer Centre (MSKCC), New York, USA in patients older than 60 years (median age 67 years) treated with BCS. At the time of surgery, HDR intraoperative radiotherapy (HDR-IORT) is delivered to the tumour bed by an iridium 192 (^192^Ir) source connected to a quadrangular silastic template applicator named Harrison–Anderson–Mick (H.A.M.^®^). A dose of 18 Gy was used since 20 Gy doses were associated with a higher rate of side effects in a prior pilot study [28]. Treatment time varies between 20 and 40 minutes. At five years (median follow-up 68 months), local recurrence rate was 7% [30], and this was similar to the rate one would expect with no radiation treatment as shown in the CALGB 9343 trial [31]. HDR-IORT is also limited by the high cost of specialised shielded operating room facilities.

Balloon catheters have been employed as intracavitary brachytherapy devices for PBI. They can be inserted into the tumour bed at the time of surgery or postoperatively. Typically, RT is delivered by balloons in 10 fractions twice a day over five consecutive days. Xoft^®^ electronic brachytherapy (eBx^®^, Xoft, Fremont, CA, USA) [32] represents the balloon device which is now being tested to deliver RT as a single fraction, totally intraoperatively, at the same dose used with the Intrabeam^®^ system (50 kpV low-energy X-rays, 20 Gy). A prospective phase-IV (post-marketing) trial is underway [33], but data are still immature, to introduce Xoft^®^ as IORT, in the current clinical practice.

## TARGIT-A trial

The TARGIT-A trial compared a strategy of a single fraction of IORT delivered using the Intrabeam^®^ system [34] to conventional 3–6 weeks of EBRT after BCS. It was a prospective phase-III trial that commenced in March 2000, enrolling 2,232 patients over 45 years old with clinically T1–T2 ≤ 3.5 cm, N0–1 invasive tumours. The pre-specified non-inferiority margin was 2.5% (80% of statistical power at 5% significance level), based on an estimated LRR of 6% with EBRT. Patients were ineligible if they were diagnosed with a lobular tumour or if they had an extensive *in situ* component. Eligible patients were treated with BCS and were randomised to either IORT or EBRT prior to surgery (pre-pathology cohort) or after surgery (post-pathology cohort). A ‘risk-adapted approach’ was used for IORT meaning that if the final pathology showed pre-specified adverse features, EBRT was administered after surgery. In these cases, IORT served as a tumour-bed boost [26].

In June 2010, the first results were published [34] with a median follow-up of 25 months, which was criticised for its shortness [35]. The LRR in the conserved breast was 1.20% (95% CI: 0.53–2.71) in the IORT arm and 0.95% (95% CI: 0.39–2.31) in the EBRT arm (p = 0.41). In order to allow accrual in subprotocols, while data matured further, the sample size was extended from 2,232 to 3,451 and recruitment continued until 2012. In November 2013, the latest results from the TARGIT-A trial were published [26], again with a short median follow-up (2 years and 4 months) due to the trial recruitment extension. The LRR with IORT was 3.3% (95% CI: 2.1–5.1%) and with EBRT, 1.3% [(95% CI: 0.7–2.5%) with a p = 0.042] but met the non-inferiority margin of 2.5%, set at the outset. Overall, breast cancer mortality in the IORT arm was 2.6% (95% CI: 1.5–4.3%) versus 1.9% [(95% CI: 1.1–3.2%) in the EBRT arm; p = 0.56] [26]. However, non-breast cancer deaths were found to be significantly reduced in the IORT arm [1.4% (95% CI: 0.8–2.5%) versus 3.5% (95% CI: 2.3–5.2%) p = 0.0086]. In terms of survival, although the log-rank statistics show a significant difference in non-breast cancer deaths that were found to be reduced in the IORT arm, these deaths also included stroke, bowel ischaemia, and other events unrelated to breast irradiation. Additionally, the median follow-up of most patients was too short to observe differences in cardiac deaths (attributable to RT) which would be expected to occur between 10 and 20 years after radiation treatment [36]. With IORT, there was a lower rate of Radiation Therapy Oncology Group (RTOG) grade 3–4 side effects and a higher rate of wound complications including seroma.

TARGIT-A trial lacks long-term follow-up data specifically on LRR. Only 1,222 patients (35%) completed 5 years of follow-up, and 611 (18%) of these received IORT. These results were more favourable for the pre-pathology stratum, and this group met the 2.5% non-inferiority margin [IORT 2.1% (1.1–4.2) versus EBRT 1.1% (0.5–2.5)], while the post-pathology did not met [IORT 5.4% (95% CI: 3.0–9.7) versus EBRT 1.7% (95% CI: 0.6–4.9; p = 0.069)]. However, the data are still too immature to draw a definitive conclusion on LR.

Cost-effective analysis is also important in view of the equipment cost and potentially small number of eligible patients at some centres. A significant investment in equipment would be required to make IORT technology available across the National Health Service (NHS) in the United Kingdom (UK) or other European countries. In United States, two cost-effective analyses have been reported [37, 38]. Alvarado *et al* concluded that when considered against the cost of EBRT, the Intrabeam^®^ system is more cost-effective [37]. It offered more quality-adjusted life years (QALYs) than the 6-week EBRT regimen. The effectiveness analysis showed that IORT was slightly preferred over the whole breast EBRT strategy when measured in QALYs (a difference of 0.00026 QALYs, or 0.95 quality-adjusted days). This result was driven by the improved utility values for the proportion of women who have salvage lumpectomy after IORT, whereas all women who undergo EBRT have a salvage mastectomy. Based on cost-minimisation analyses by Shah *et al*, IORT represents a potential cost savings in the management of early-stage breast cancer. However, absolute reimbursement is misleading, because when additional medical and non-medical costs associated with IORT are factored in, EBRT represents the cost-effective modality based on cost per QALY analyses [38].

## ELIOT trial

The ELIOT trial recruited its first patients in November 2000 at the European Institute of Oncology (EIO) in Milan (Italy). It was a prospective single-centre randomised phase-III equivalence trial. The aim of this trial was to compare a 21 Gy single-dose IOERT delivered using the ELIOT technique [39] to conventional whole breast EBRT. The pre-specified equivalence margin was 7.5% (90% of statistical power at 5% significance) and a non-inferiority margin of 4.5%. A total of 1,305 women were randomised, aged between 48 and 75 years with clinically invasive T1–T2 ≤ 2.5 cm breast cancers suitable for BCS. Tumours, which were either ductal or lobular carcinoma, *in situ* were excluded in this study. A diagnosis of invasive lobular carcinoma did not affect eligibility. The trial was closed for recruitment in December 2007. The LRR was found to be 4.4% (95% CI: 2.7–6.1) in the ELIOT arm and 0.4% (95% CI: 0.0–1.0) in the EBRT arm (p = 0.0001) [27], at 5.8 years median follow-up. The LRR just fell within the pre-defined non-inferiority margin of 4.5%. The hazard ratio (HR) for LRR in the ELIOT trial was 9.3 (95% CI: 3.3–26.3) for women allocated to receive IOERT compared with those allocated to receive EBRT [27]. It was found that there was no significant difference in the 5-year overall survival rate in two arms, that is, 96.8% (95% CI: 95.3–98.3) in the ELIOT arm and 96.9% (95% CI: 95.5–98.3) in the EBRT arm (p = 0.59) [27].

The ELIOT trial included more than 10% of patients with worse prognosis (larger tumours, nodes positive) which may account for the increased LRR observed with IORT. In the multivariable analysis, carcinoma greater than 2 cm and poorly differentiated, more than four positive nodes and triple negative subtypes resulted in doubling the risk of LRR. Consistent with a low-risk group, showing 5 years occurrence of 1.5% LR (p < 0.0001) emerged as a key finding in the ELIOT population. Moreover, a noteworthy retrospective analysis of 1,822 patients treated with the ELIOT technique IORT off-trial at EIO was published, after American Society for Radiation Oncology (ASTRO) and Groupe Europèen de Curietherapie of the European Society of Therapeutic Radiology and Oncology (GEC-ESTRO) recommendations were made available in 2009. Patients who fit the ASTRO ‘suitable’ group guidelines and the GEC-ESTRO ‘low-risk’ group guidelines, were found to have a LRR of 1.5% and 1.9%, respectively [40, 41], confirming that IORT should be restricted to selected patients on the basis of tumour characteristics.

In terms of cost-effectiveness, despite the higher initial device-related costs, the cost per patient treated with IORT by using electron beams linear accelerators is effective when considering the reduction in radiotherapy waiting lists, pre-treatment planning and delay, but a specific cost-effective analyses is required, since IORT devices remain unaffordable for many hospitals worldwide [42].

## ASTRO and GEC-ESTRO recommendations in 2009

ASTRO and GEC-ESTRO recommendations have been drawn up to give clinicians a pragmatic approach to PBI. The ASTRO panel grouped patients into suitable, cautionary, and unsuitable for PBI on the basis of a variety of diseases and clinical characteristics [43]. The GEC-ESTRO panel stratified patients undergoing PBI into low, intermediate, and high-risk categories [44]. ASTRO and GEC-ESTRO recommendations differ in terms of patients’ age, tumour size, and oestrogen receptor status among the lowest risk groups. However, neither ASTRO nor GEC-ESTRO guidelines refer specifically to IORT. The Italian retrospective analysis of ELIOT technique showed that the 5-year LRR increased as patients moved from ASTRO ‘suitable’ to ‘cautionary’ to ‘unsuitable’ groups (1.5%, 4.4%, and 8.8%, respectively), while the 5-year LRR for ‘low-risk’, ‘intermediate-risk’, and ‘high-risk’ groups according to the GEC-ESTRO guidelines was 1.9%, 7.4%, and 7.7%, respectively. The slightly higher risk of recurrence in the ‘low-risk’ and ‘intermediate-risk’ groups from GEC-ESTRO might be due to the differences in selection criteria mentioned previously. Since IORT techniques are increasingly being used worldwide, specific guidelines on IORT are needed.

## Clinical application of TARGIT-A and the ELIOT trials

The TARGIT-A and ELIOT trials demonstrated that IORT and IOERT are not inferior to EBRT in selected patients [45, 46]. A subset of low-risk women for whom IOERT is acceptable in terms of LRR at 5.8 years of median follow-up has emerged from the ELIOT trial. Data from the TARGIT trial are encouraging and promising at 29 months of median follow-up with IORT, but longer follow-up is required. Data from totally intraoperative intracavitary balloon application by Xoft technology are still immature, while HDR-IORT demonstrated that ASTRO recommendations are ineffective for patients treated with this approach. Moreover, Intrabeam^®^ and both Liac^®^ and Novac7^®^ devices employed in these trials have been validated, whereas it is not clear the level of clinical evidence of the rest of novel devices introduced into the market.

However, there are some clear advantages of single-fraction RT for patients, and the level of evidence available on IORT is significant enough to share with them. Patients deemed at low risk of LR or those deemed suitable for PBI, according to the GEC-ESTRO and ASTRO recommendations, could be considered as candidates for IORT delivered under strict protocol, even though more specific guidelines for IORT would be helpful to assist clinicians in patient optimal selection. Both TARGIT-A and ELIOT techniques have been evaluated within phase-III trials launched over 14 years ago, and although further follow-up will increase confidence with the data, it will also further delay clinical implementation. National registries should be set up in order to monitor LR events occurring after IORT treatments off-trial, prospectively and might be useful to accumulate data.

## Conclusion

In conclusion, in light of the existing data, IORT and IOERT should be now considered as an alternative to EBRT for specifically selected and well-informed patients. The higher risk of local recurrence should be widely discussed and compared with the great advantage of a single-fraction radiation treatment. The current paradigm is that of striving to deliver ‘minimum effective treatment’ to patients with cancer, and with this in mind, it is necessary to inform suitable patients about the novel options for the treatment of their cancer.

## Conflicts of interest

The authors have no disclosures to make concerning financial and personal relationships with other people or organisations that could inappropriately influence their work.
